# PyRMD Studio: A Unified Suite for Next-Generation, AI-Powered Virtual Screening

**DOI:** 10.1021/acs.jcim.6c00648

**Published:** 2026-05-19

**Authors:** Benito Natale, Muhammad Waqas, Michele Roggia, Salvatore Di Maro, Sandro Cosconati

**Affiliations:** DiSTABiF, University of Campania Luigi Vanvitelli, Caserta 81100, Italy

## Abstract

Artificial Intelligence (AI) has become a cornerstone of modern drug discovery. Yet, its widespread adoption is often hindered by the steep learning curve of command-line tools and the prevalence of overfitting due to improper data splitting. Here, we introduce PyRMD Studio, a major update to our open-source virtual screening (VS) software, designed to address these challenges through enhanced accessibility, scientific rigor, and computational efficiency. This new version features a comprehensive graphical user interface (GUI) compatible with both Linux and Windows, democratizing access to sophisticated AI workflows and allowing nonexpert users to perform both ligand-based and structure-based VS without coding expertise. To ensure reliable performance estimation, PyRMD Studio implements a rigorous validation strategy based on Butina clustering, which separates structural clusters between training and test sets to mitigate data leakage and prevent overoptimistic benchmarking. Furthermore, extensive code optimizations and vectorization have resulted in an approximate 3.3-fold increase in screening speed compared to the previous release. By combining a user-friendly environment with robust validation protocols, PyRMD Studio provides a streamlined and reliable toolkit for high-throughput virtual screening campaigns. PyRMD Studio is freely available at https://github.com/sandrocosconati/PyRMD-Studio.

## Graphical abstract

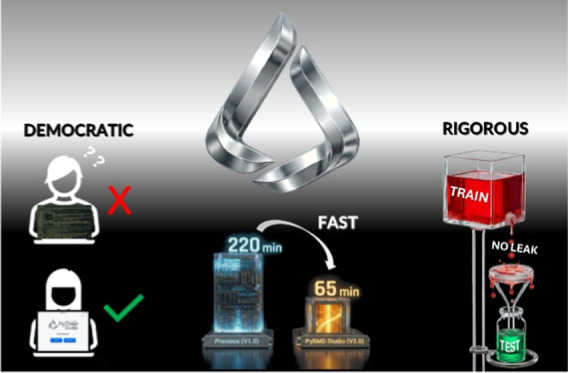

## Introduction

The integration of artificial intelligence (AI) into the drug discovery pipeline has fundamentally shifted the paradigm of virtual screening (VS), offering unprecedented speed and predictive power in the identification of novel bioactive compounds.^1^ However, despite the proliferation of sophisticated machine learning (ML) and deep learning (DL) algorithms, a significant barrier remains between state-of-the-art computational methods and the medicinal chemists who could benefit from them most. Many powerful open-source tools rely heavily on command-line interfaces (CLIs) or require complex coding environments, effectively excluding nonexpert users from leveraging these technologies independently.

In our previous work, we introduced PyRMD,^2^ a fully automated Python-based tool implementing the random matrix discriminant (RMD) algorithm. Designed to be trained on bioactivity data directly from ChEMBL,^3^ PyRMD demonstrated robust performance in ligand-based virtual screening (LBVS) and was subsequently integrated into the PyRMD2Dock^4^ protocol to streamline large-scale Structure-Based Virtual Screening (SBVS) campaigns. Interestingly, PyRMD has already been successfully used for real-world VS campaigns.^5–8^ However, despite automating much of the data curation and training process, PyRMD still primarily operates as a CLI tool, thus requiring users to manually navigate configuration files and terminal commands.

Beyond accessibility, a second critical challenge in contemporary AI-driven drug discovery is the reliability of performance estimates. Recent discourse in the cheminformatics community has highlighted the prevalence of “data leakage” in benchmarking, a phenomenon where structural redundancy between training and test sets leads to inflated performance metrics that fail to translate into prospective screening success. Standard random splitting methods are nowadays known to usually fail in separating distinct chemical series, causing models to “memorize” structural motifs rather than truly learn generalizable rules of molecular recognition.

To address these dual challenges of accessibility and rigorous validation, we present PyRMD Studio. This major update introduces a comprehensive graphical user interface (GUI), transforming the software into an intuitive desktop suite accessible to researchers without coding expertise. Furthermore, PyRMD Studio implements a rigorous data splitting strategy based on Butina clustering to mitigate data leakage, ensuring that performance benchmarks provide a realistic estimate of the model’s generalization capability. Coupled with significant code optimizations that increase screening speed by over 3-fold when compared to the previous version, PyRMD Studio represents a robust, transparent, and user-friendly solution for modern virtual screening campaigns. The suite is structured to work as a LBVS tool, which accepts ChEMBL IDs or simple SMILES CSV files as input, and a SBVS tool, utilizing CSV files with external docking scores. Ultimately, the software enables the efficient screening of ultralarge chemical libraries in a reasonable amount of time (e.g., 1 million compounds in ∼60 min).

### Overview of PyRMD Studio

One of the main issues usually faced in the artificial intelligence (AI) field is the way the initial data set is split into training and test sets. As recently outlined by Jain et al.^9^ and further discussed in a blog post,^10^ very common practices in doing so, like random split, result in the test set containing molecules that too closely resemble those in the training set. This phenomenon, known as data leakage, can severely compromise the reliability of model evaluation by leading to an overly optimistic assessment of its performance and, ultimately, model overfitting. Specifically, the consequence of data leakage is that the model learns to recognize specific examples rather than generalizable patterns, making its predictive capabilities on truly novel data unreliable. To effectively mitigate this critical issue in our benchmarking experiments, the new version of PyRMD (namely, PyRMD Studio) directly implements a cross-validation adapted version of the StratifiedGroupKFold splitting utility, coupled with Butina clustering. Specifically, by grouping molecules with the Butina algorithm and then splitting them into train and test sets with a stratified approach, this robust method ensures that no examples from the same cluster are present in both the training and test set splits for all the K cross-validation rounds repeated n times. This proactive measure effectively prevents data leakage, thereby enabling a more accurate and realistic evaluation of the generalization capabilities of our models.

While retaining the same model architecture of the original PyRMD, the new version, PyRMD Studio, also incorporates some code refinements and features specifically designed to speed up the overall calculation time without altering the underlying core random matrix discriminant (RMD) algorithm.^11^ These enhancements are particularly evident in the two key operational modes.

1. In the Benchmarking mode, several optimizations have been introduced. Specifically, internal “for loops” within critical functions have been refactored into their faster, vectorized equivalents, significantly reducing processing time. Furthermore, clustering results are now strategically saved as temporary files, which allows for much faster re-execution of clustering rounds when required, streamlining repetitive analyses.
2. For the Screening mode, efficiency has been enhanced by implementing the automatic saving of fitted model hyperparameters to disk. This crucial improvement eliminates the need to refit the chosen model repeatedly, drastically cutting down on setup time.

These systematic optimizations have led to a notable overall reduction in computational time. For instance, we screened 1 million molecules with a model trained on FGFR1 (the same data set employed for testing the previous PyRMD version’s processing time, as detailed in our previous paper). Results indicate an average speed-up of approximately 3.3-fold (+330%) when compared to the previous version (Figure 1). To ensure a rigorous and reproducible comparison with our previous publication, this primary benchmark was executed on identical high-performance hardware. However, it is worth noting that we also successfully evaluated the same 1 million compound screening on a standard consumer laptop (AMD Ryzen 7 5700U, 16 threads, 8 GB RAM). These qualitative tests confirmed that PyRMD Studio runs efficiently on everyday hardware, effectively bypassing the need for high-performance clusters for routine screening applications. This substantial efficiency gain significantly improves the usability of PyRMD Studio, enabling more extensive and rapid exploration of chemical space.

**Figure 1:**
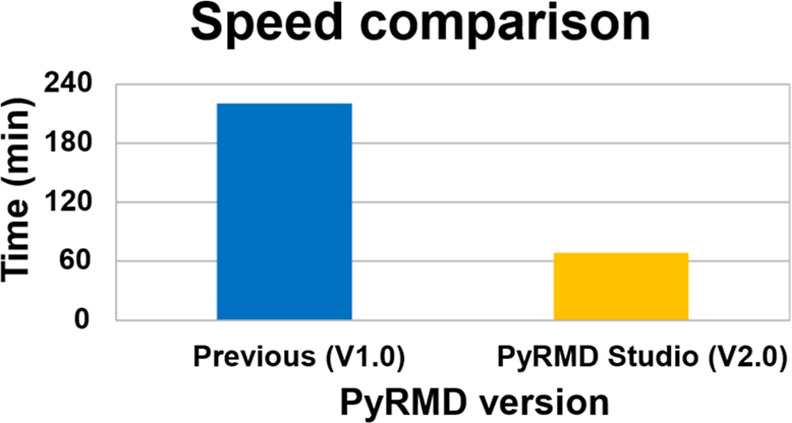
Time required (in minutes) to perform the screening of 1 million molecules by the previous PyRMD (blue) and the new PyRMD Studio versions (orange). Both experiments were performed on an Intel Xeon Gold 6238R CPU (112 threads total, 754 GB RAM).

Ultimately, PyRMD Studio is now embedded within a user-friendly Graphical User Interface (GUI) that acts as a suite for both PyRMD, our Ligand-based Virtual Screening (LBVS) tool, and PyRMD2Dock, our Structure-based Virtual Screening (SBVS) tool. Our core motivation behind the development of this GUI is to further democratize the nowadays acknowledged power of AI in real-life LBVS and SBVS campaigns, making it accessible to non-CLI-expert users. The proposed GUI has been meticulously tailored to manage all preprocessing and calculation tasks with just a few mouse clicks, offering a “Non-Expert Mode” that requires no prior knowledge of the underlying AI theory. Complementary to this, the presence of an “Expert Mode” provides advanced users with the flexibility to unlock a deep fine-tuning of the model, catering to more specialized research needs.

### GUI Workflow and Modules

The PyRMD Studio interface is designed to guide the researcher through the standard virtual screening pipeline via a logical, tab-based organization (Figure 2). Upon launching the application, the main dashboard allows the user to select between two primary operational modules: **PyRMD** for Ligand-Based Virtual Screening (LBVS) and **PyRMD2Dock** for Structure-Based Virtual Screening (SBVS).

**Figure 2:**
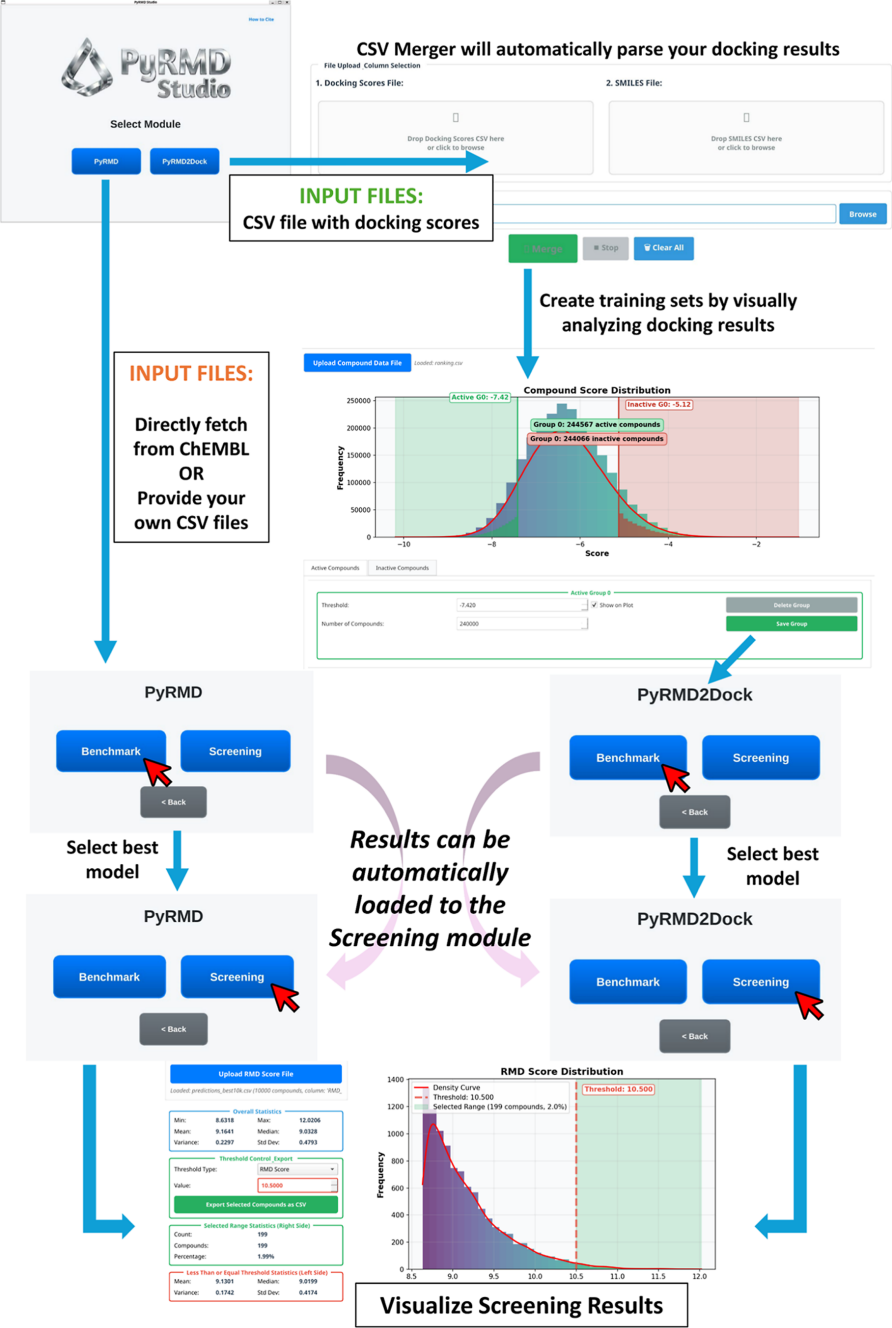
Overview of the PyRMD Studio workflow.

#### Benchmarking

In the Benchmarking tab (Figure S1), the software simplifies data curation depending on the chosen strategy. For **LBVS (PyRMD)**, the workflow is streamlined to handle preclassified bioactivity data: users simply upload separate lists for active and inactive compounds or fetch them directly from ChEMBL by entering the target ChEMBL ID.

Conversely, the **SBVS module (PyRMD2Dock)** offers various options in data input: users can either process raw outputs from docking software through an automated parsing utility (“CSV merger”) or directly upload preprocessed docking data sets (as CSV files). In this specific case, an integrated “Analyze” function (referred as “Compound Distribution Analyzer”, Figure S2) is also present, which permits the visualization of the docking score distribution of these data sets, and so allows researchers to interactively define the activity thresholds (e.g., via sliders) that will generate the final training sets for the RMD algorithm.

However, for both modules the interface provides critical hyperparameters (e.g., Active/Inactive Epsilon Cutoffs) and other required parameters/files with tooltips, while the “Expert Mode” further grants access to advanced settings like inhibition thresholds, statistical parameters and fingerprint customization. A key feature of this phase is the “Update Configuration” function, which serializes the selected parameters into backend configuration files, ensuring reproducibility. Furthermore, users can use the “Open Results CSV” function to upload precalculated PyRMD Studio benchmarking results, allowing them to proceed directly with the Screening phase.

#### Screening and Analysis

Once a model is trained and validated, the workflow transitions to the Screening tab. Here, the user selects the best-performing model previously identified and automatically saved during the benchmarking phase and applies it to large external databases. Finally, the results can be processed in the result analysis module (see Figure 2). This interactive tool moves beyond simple CSV outputs by visualizing the score distribution of the screened library, allowing users to dynamically apply thresholds and visualize the separation between potential hits and the background noise, facilitating a more informed selection of candidates for experimental validation. For detailed step-by-step tutorial and installation instructions, as well as example CSV files and tutorials, users are referred to the full documentation available on our GitHub repository.

### Implementation

PyRMD Studio was developed using Python 3, designed for compatibility on Ubuntu and Windows Subsystem for Linux. The new graphical user interface (GUI) was built using the PyQt5 ^12^ package of Python, ensuring accessibility for nonexpert users without requiring command-line interaction. For core data processing and numerical calculations, the software relies on the scientific Python ecosystem, including NumPy^13^ and pandas;^14^ notably, critical routines from the previous version were refactored into vectorized operations using these libraries to achieve an average speed-up of 3.3× in calculation time. The cheminformatics capabilities, including SMILES standardization, fingerprint generation (e.g., MHFP6), and the newly implemented Butina clustering for data leakage mitigation, are powered by RDKit.^15^ Machine learning operations utilize scikit-learn.^16^ Plots are rendered via matplotlib^17^ and seaborn.^18^ The software is distributed as an open-source tool available on GitHub, with environment management facilitated via Anaconda^19^ to ensure consistent dependency resolution across different operating systems (Windows, Ubuntu Linux). The installation process is fully automated via a single file, which automatically handles the setup of the conda environment, if not already present, and configures it, allowing users to deploy the software with minimal effort.

### Benchmarking Experiments

To analyze the performance of the newly implemented Butina clustering in effectively reducing the data leakage phenomenon, PyRMD Studio was benchmarked against the same set of targets employed for the original PyRMD version’s benchmarking experiments, as detailed in our previous publication.^2^ Specifically, we conducted experiments using identical data sets and default hyperparameters (for a detailed explanation of the specific hyperparameters required, please refer to our previous papers). These settings included 0.84, 0.95, and 0.98 as *epsilon_cutoff_active* values, and 0.7, 0.84, 0.95, and 0.98 as *epsilon_cutoff_inactive* values. For each target, we considered the benchmarking results generated by the optimal *epsilon_cutoff_active*/*epsilon_cutoff_inactive* combination in terms of the best F-score and compared these against the results from the previous PyRMD version (Figure 3A).

**Figure 3:**
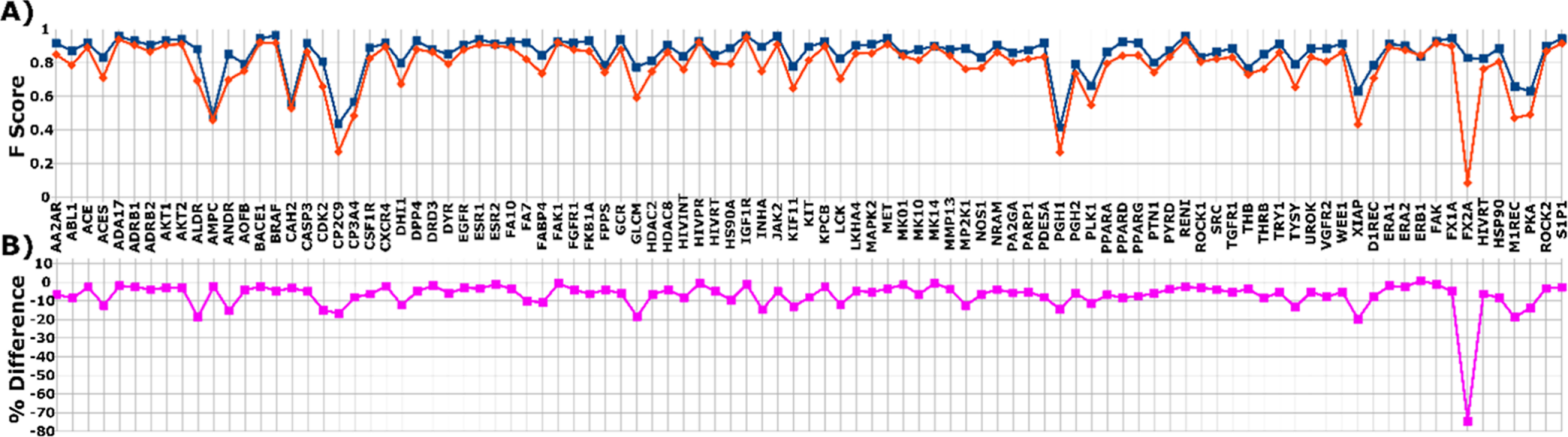
(A) performance analysis of the *F*-score metric for different data sets, comparing the previous PyRMD (blue) with the new PyRMD Studio versions (orange) in benchmarking experiments. (B) performance difference between the previous PyRMD and the new PyRMD Studio versions, calculated as the relative percentage between the respective *F*-score values for all the data sets.

Overall, we can genuinely conclude that the benchmarking results of PyRMD Studio are comparable to those of the previous version, with an average *F*-score reduction of ∼9% (Figure 3B). This slight decrease in performance can be ascribed to the repeated StratifiedGroupKFold splitting (5 folds, 3 reps) of the data set previously clustered with the Butina algorithm. Conversely, the previous PyRMD version, which employed a standard Repeated Stratified K-Fold cross-validation^20^ approach (5 folds, 3 reps), did not entirely prevent the leakage of highly similar compounds into the test set. This led to a detectable overestimation of the model’s predictive capability in the previous version, making its reported higher performance less indicative of true generalization. The modest reduction in *F*-score on average for all the data sets observed with PyRMD Studio, therefore, reflects a more realistic and robust assessment of its performance on truly novel chemical space.

It is worth noting that PyRMD Studio, when compared to its previous version, generally performed less optimally on targets characterized by a high degree of structural heterogeneity within the active/inactive compound sets, or on those with very limited numbers of experimentally confirmed active molecules. This behavior is likely due to the more stringent separation enforced by the Butina clustering, which, while preventing data leakage, might sometimes remove structurally unique but genuinely active compounds from the training set, making it harder for the model to learn. Conversely, PyRMD Studio performed better or comparably on targets with well-defined structure–activity relationships in the active/inactive sets, where the rigorous clustering strategy effectively prevented the model from overfitting to specific structural motifs. A striking illustration of this phenomenon is the FX2A data set, a notable outlier in our benchmarking that exhibited a significant *F*-score reduction compared to the previous version. As shown in the chemical space analysis (Figure S3), ∼65% of the active compounds in this data set belong to a single, dense cluster. While random splitting previously allowed this dominant scaffold to be shared between training and test sets (causing data leakage and overoptimistic performance), PyRMD Studio enforces a strict separation. Consequently, the model must generalize to diverse scaffolds or predict a completely novel chemical series, leading to a more realistic, albeit lower, performance metric.

We also compared the benchmarking performance of PyRMD Studio with that of Random Forest models (Figure S4), which are generally recognized as robust to noise to some extent and capable of capturing complex nonlinear relationships. Overall, our findings with PyRMD Studio underscore the critical importance of careful data splitting methodologies in accurately assessing model performance in virtual screening campaigns, a challenge that even highly performant models like Random Forest must contend with, if not properly addressed.

### Limitations

The PyRMD Studio GUI is designed to democratize access to rigorous AI-driven screening, enabling users with limited coding experience to perform complex LBVS and SBVS campaigns. However, users should be aware of specific methodological and technical constraints. A primary consideration is the impact of the newly implemented “antileakage” splitting strategy; while Butina clustering with a 0.65–0.70 default similarity threshold ensures realistic performance estimates, it may yield lower performance metrics on targets characterized by very high structural heterogeneity or limited data availability, as rigorous clustering can exclude unique active scaffolds from the training process. Therefore, for targets with sparse bioactivity data, users are advised to utilize the “Expert Mode” to manually tune clustering thresholds or revert to standard splitting if necessary. Also, we recommend a minimum of 100 compounds for both the active and inactive training sets for optimal performance.

From a computational standpoint, the underlying Butina clustering scales quadratically (O­(*n* ^2^)). While default activity thresholds effectively filter massive data sets to manageable sizes, clustering excessively large, unfiltered data sets will cause severe bottlenecks. Future updates aim to integrate scalable alternatives like the BitBIRCH algorithm.^21,22^ Additionally, during the screening phase, although the core RMD algorithm has been optimized via vectorization to achieve significant speed-ups, the analysis of ultralarge chemical libraries (e.g., >100 million compounds) remains memory-intensive. Users may encounter system slowdowns depending on available RAM and the dimensionality of the selected fingerprints. To mitigate this, PyRMD Studio includes automated chunking routines, though users are encouraged to filter databases by physicochemical properties before screening to optimize throughput. Finally, the PyRMD2Dock module relies on external third-party software, specifically docking softwares, for the structure-based validation step. Consequently, the execution of confirmation docking runs is subject to the successful installation of these programs, which are outside the scope of the PyRMD Studio Python environment.

## Conclusions

The democratization of AI in drug discovery requires tools that are not only powerful but also accessible and scientifically rigorous. With PyRMD Studio, we have significantly advanced the usability of the RMD algorithm by wrapping its complex workflows into a user-friendly Graphical User Interface. This development empowers wet-lab scientists and nonexpert users to perform sophisticated Ligand-Based and Structure-Based Virtual Screening campaigns without the steep learning curve associated with command-line environments.

Crucially, PyRMD Studio addresses the pervasive issue of data leakage in machine learning benchmarks. By integrating Butina clustering into the validation routine, the software enforces a strict structural separation between training and test sets. While this rigorous approach may yield slightly lower absolute performance metrics compared to random splitting, it provides a far more honest and realistic assessment of a model’s predictive power on truly novel chemical scaffolds. This shift toward “realistic” rather than “optimistic” benchmarking is essential for increasing the success rate of prospective screening campaigns.

Furthermore, the code optimizations introduced in this version have resulted in a dramatic reduction in computational overhead, enabling the screening of millions of compounds on standard workstations in a fraction of the time previously required. By combining the speed of the RMD algorithm, the rigor of cluster-based validation, and the accessibility of a GUI, PyRMD Studio stands as a comprehensive toolkit for streamlining the hit identification process. We believe this updated suite will facilitate broader adoption of AI methods in academic and industrial settings, ultimately accelerating the discovery of novel therapeutic agents.

## Supplementary Material















## Data Availability

PyRMD Studio is freely available at https://github.com/cosconatilab/PyRMD-Studio.

## Notes

List of ChEMBL data sets sets used (ZIP) (ZIP)
List of decoys data sets used (ZIP) (ZIP)
The following files are available free of charge. Additional Figures (PDF) (PDF)
Information regarding the ChEMBL IDs of the targets employed in the benchmark tests (XLSX) (XLSX)
Benchmarking results of the previous PyRMD version (XLSX) (XLSX)
Comparison of the benchmarking results between the previous PyRMD version and the new PyRMD Studio version (XLSX) (XLSX)
Comparison of the F scores between a Random Forest model and PyRMD Studio (XLSX) (XLSX)

## Abbreviations

AI: artificial intelligence
VS: virtual screening
GUI: graphical user interface
ML: machine learning
DL: deep learning
CLI: command-line interface
RMD: random matrix discriminant
LBVS: ligand-based virtual screening
SBVS: structure-based virtual screening
