# Supplementary material for: PyRMD Studio: A Unified Suite for Next-Generation, AI-Powered Virtual Screening
Source: J Chem Inf Model. 2026 May 19;66(11):6197–203. doi: 10.1021/acs.jcim.6c00648 (PMC13250901; doi:10.1021/acs.jcim.6c00648)
Supplement: Supplementary file 3 [file ci6c00648_si_003.pdf]

Supporting Information

PyRMD Studio: A Unified Suite for Next-Generation, AI-Powered Virtual Screening

*Benito Natale,‡ Muhammad Waqas,‡ Michele Roggia, Salvatore Di Maro, Sandro Cosconati**

DiSTABiF, University of Campania Luigi Vanvitelli, 81100 Caserta, Italy

CONTENTS

Figures S1-S4 **S2-4**

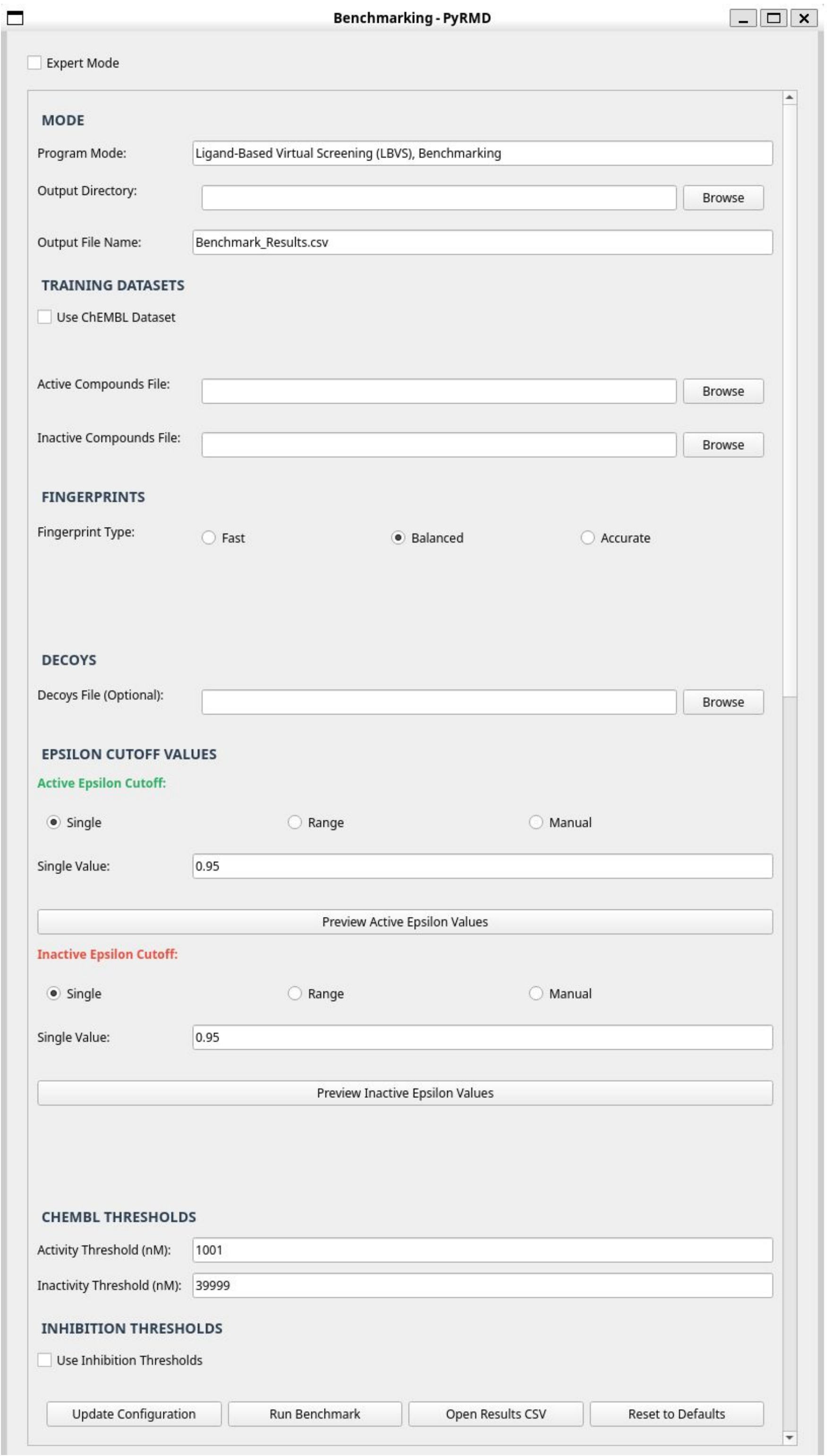
Benchmarking - PyRMD
Expert Mode
MODE
Program Mode:
Ligand-Based Virtual Screening (LBVS), Benchmarking
Output Directory:
Browse
Output File Name:
Benchmark_Results.csv
TRAINING DATASETS
Use ChEMBL Dataset
Active Compounds File:
Browse
Inactive Compounds File:
Browse
FINGERPRINTS
Fingerprint Type:
Fast
Balanced
Accurate
DECOYS
Decoys File (Optional):
Browse
EPSILON CUTOFF VALUES
Active Epsilon Cutoff:
Single
Range
Manual
Single Value:
0.95
Preview Active Epsilon Values
Inactive Epsilon Cutoff:
Single
Range
Manual
Single Value:
0.95
Preview Inactive Epsilon Values
CHEMBL THRESHOLDS
Activity Threshold (nM):
1001
Inactivity Threshold (nM):
39999
INHIBITION THRESHOLDS
Use Inhibition Thresholds
Update Configuration
Run Benchmark
Open Results CSV
Reset to Defaults

Figure S1. Benchmarking configuration screen with default values in “Non-Expert Mode”.

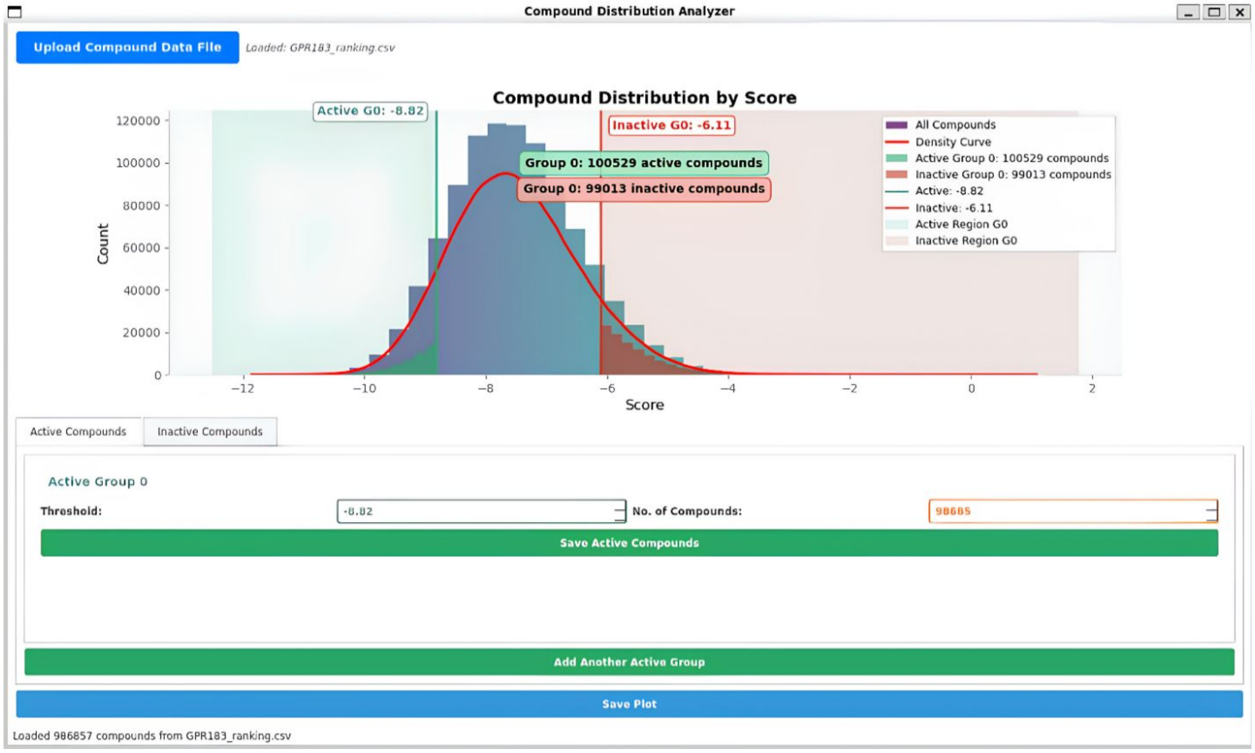

Figure S2. Compound Distribution Analyzer is one of the utilities in PyRMD Studio that allows the user to analyze the docking score distribution and select the “actives” and “inactives” compounds to be used as training data.

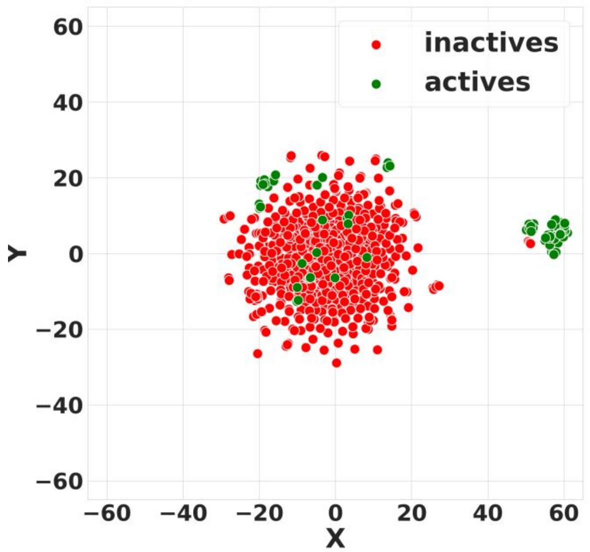

Figure S3. t-SNE visualization of the FX2A dataset's chemical space.

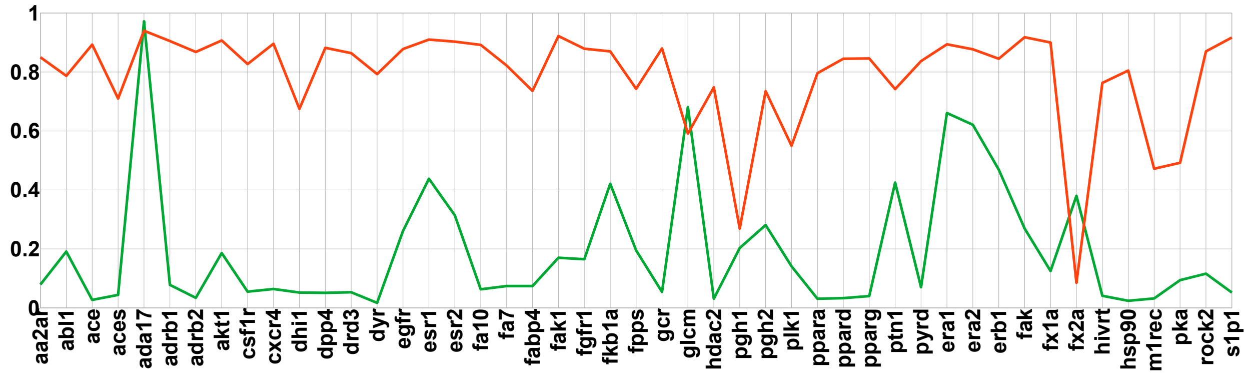

Figure S4. F-score comparison in benchmarking experiments between Random Forest (green) and RMD (orange) algorithms.
